# Assessing fecal pollution source in a Northern Michigan Lake using qPCR and a community-based monitoring framework

**DOI:** 10.1371/journal.pone.0331494

**Published:** 2025-08-29

**Authors:** Kelsey L. Froelich, Ronald L. Reimink, Ceilidh P. Welch, John Ransom, Simon J.G. Otto, Patrick C. Hanington

**Affiliations:** 1 School of Public Health, University of Alberta, Edmonton, Alberta, Canada; 2 Freshwater Solutions, LLC, Cedar, Michigan, United States of America; 3 Benzie County Conservation District, Benzie County, Michigan, United States of America; 4 Healthy Environments, Centre for Healthy Communities, School of Public Health, University of Alberta, Edmonton, Alberta, Canada; Retired-United States Environmental Protection Agency, UNITED STATES OF AMERICA

## Abstract

Implementing quantitative polymerase chain reaction (qPCR) within a community-based research framework expands the scope and scale of community-driven monitoring and research efforts. The increasing accessibility of qPCR technology and methodology has allowed the incorporation of community partners in numerous ways, ranging from sample collection to running qPCR tests. Here, we report on a community-driven study at Crystal Lake in Beulah, MI, in which qPCR was demonstrated to be a more valuable water testing technique than culture-based methods. Historically high levels of the enteric bacteria *Escherichia coli* in the inlet to Crystal Lake, Cold Creek, as measured by culture-based testing methods, spurred an interest in understanding more about fecal pollution and its source. In this study, we monitored 17 sites in Cold Creek and around Crystal Lake throughout the summers of 2020 and 2021 and used qPCR to assess levels of *Enterococcus* while source-tracking all samples for human, dog, and Canada goose fecal markers (HF183, DG3 and CG0F1-Bac, respectively). Replicate samples were sent for *E. coli* culture-based testing. Results showed high fecal contamination (*E. coli* and *Enterococcus*) and consistent HF183, DG3 and CG0F1-Bac-positive samples at specific sample sites. Varying degrees of relatedness were found between *Enterococcus* levels grouped by precipitation amount. Due to the nature of the sampling sites, we hypothesize that human fecal contamination is due to stormwater outflows and septic system influences and not direct human contact with the water. A Cohen’s Kappa analysis between the *Enterococcus* qPCR test results and *E. coli* culture-based test results indicated a moderately positive relationship. The historical *E. coli* dataset, now accompanied by the *Enterococcus*, HF183, DG3 and CG0F1-Bac data, confirms consistent and elevated levels of fecal pollution in Cold Creek and Crystal Lake that is likely related to human sources with stormwater outflows being a contributor to this contamination.

## Introduction

Community-based monitoring (CBM) is a relatively new but widely used tool for freshwater studies worldwide [1]. Also called “citizen science,” using ideas and resources from engaged community members can have far-reaching impacts compared to a small group of scientists [2]. CBM has enhanced many aquatic biology initiatives, including assessments of ecosystem health, macroinvertebrate classification, and parasite diversity [3–5]. The range of community involvement in CBM projects can vary substantially, sometimes including high levels of engagement from community partners in study development and data collection [1]. In this study, local community members approached scientists with concerns about the impact of fecal bacteria on water quality, and a research approach was designed around their concerns.

Fecal indicator bacteria (FIB) can indicate the presence of disease-causing pathogens in the water due to or associated with fecal contamination [6]. *Escherichia coli* and *Enterococcus* are standard FIB recommended by the US Environmental Protection Agency (EPA) [7] as targets for water quality testing. *Enterococcus* can be assessed using traditional plating methods or via quantitative polymerase chain reaction (qPCR), while *E. coli* is frequently assessed using plating or most probable number (MPN)-based methods per the EPA guidelines for recreational and wastewater testing [7,8]. Culture-based plating methods and MPN-based methods require a 24-hour and 18–24-hour completion time, respectively and bacteria to be viable and actively growing for detection. In contrast, qPCR methods are faster (less than three hours), can detect DNA from live or dead organisms or environmental (e)DNA, and can be used to determine the source(s) of detected FIB contaminants in water samples via microbial source tracking [9]. Recently, the EPA has provided guidance for sampling *E. coli* via qPCR [10]. A statistical analysis study conducted by Gonzalez and Noble (2014) comparing qPCR to culture-based water testing methods showed that using culture-based methods correctly predicted management decisions at a slightly higher rate than qPCR [11]. However, Wade et al. (2022) [12] found that *Enterococcus* measured by qPCR more accurately predicted GI illness in children than membrane filtration methods [13].

From the perspective of a community-driven project, qPCR confers advantages over a culture-based methodology. Filtration of a composite water sample and preservation of the filter for future qPCR analysis is more accessible than the parallel process for culture analysis [14]. qPCR analysis can even be made accessible to community partners, decentralizing the equipment required away from core laboratories and using relatively inexpensive, portable machines. This allows for more water samples to be taken in each area or time period and gives communities more control over the type of questions with which to engage [15].

Another significant benefit of using qPCR as the method for quantifying fecal contamination is the ability for microbial source tracking. While *E. coli* and *Enterococcus* are commonly used indicators of fecal pollution, they are nonspecific to their source. Microbial source tracking (MST) can be performed using qPCR to identify the source(s) of the fecal contamination. Of interest in this study is the contribution of human, dog, and Canada goose fecal pollution to the overall fecal contamination measured at a lake. The HF183 MST marker that targets human *Bacteroides* is used as part of a specific and reliable qPCR-based assay to measure human fecal pollution [16,17]. Dogs and Canada geese are also common in our study area and have MST markers that are reliably tested have historical use (DG3 and CG0F1-Bac, respectively) [18,19].

Stormwater sewer systems have been shown to carry high loads of FIB [20–23], including human fecal material [24–28], while in Michigan, fecal pollution of human origin is an issue that has been linked to the density of septic systems [29]. Fecal contamination around the Village of Beulah and Cold Creek in Crystal Lake, MI, has been a growing concern of residents of the Betsie River/Crystal Lake watershed [30]. The Benzie/Leelanau Health Department has been monitoring Beulah Beach near the outlet of Cold Creek since 2013 and has closed the beach for full body contact for 20 days from 2013–2022 [31]. The Crystal Lake Watershed Association (CLWA) and Benzie Conservation District (BCD), both primary caretakers of the Crystal Lake Watershed, have invested resources to collect and analyze water samples for enteric bacteria in the Cold Creek watershed annually since 2016. Before that, samples were analyzed periodically by the State of Michigan.

In this study, we sampled at critical points along Cold Creek and near its mouth on Crystal Lake. Samples were analyzed using qPCR targeting *Enterococcus*, followed by source tracking all samples for human, dog and Canada goose fecal contamination (HF183, DG3, and CG0F1-Bac markers, respectively). Additionally, we compared qPCR results assessing *Enterococcus* with traditional plating techniques testing for *E. coli*. By working with community partners to answer a question of interest for them, we exemplify the power of community-based monitoring to increase water quality interest by the public and show the benefit of using qPCR as opposed to culture-based water testing methods, to further test water samples and clarify questions about the source(s) of fecal contamination.

## Methods

### Ethics statement

Ethics approval for this study was waived by the University of Alberta Research Ethics Board. It was determined that this study meets one of the conditions outlined under Chapter 2 of the Tri-Council Policy Statement: Ethical Conduct for Research Involving Humans – TCPS 2 (2022) as an activity that does not require Research Ethics Board review. No human subjects or data associated with human subjects or animals were involved in this research.

### Sample locations

**Fig 1:**
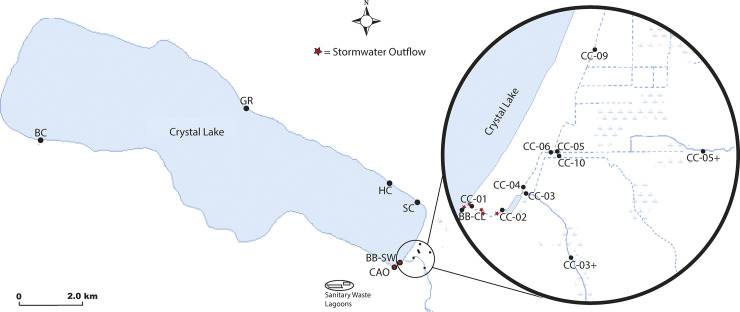
Map of sampling locations around Crystal Lake, Benzie County, MI. Zoomed image shows sampling sites along Cold Creek inlet. Fifty mL of water was collected at each site throughout the summers of 2020 (weekly) and 2021 (biweekly) and tested for *Enterococcus* and *E. coli*. Blue lines represent waterbodies and relevant inflows. Dots represent sample sites. Stars indicate stormwater outflow locations near or corresponding to our sampling sites. Basemap courtesy of the U.S. Geological Survey.

A map outlining the sampling sites for this project is presented in Fig 1. Sampling took place in the Betsie River/ Crystal Lake Watershed, which spans 242 square miles in northern Michigan. Most of the sampling took place in the Village of Beulah, located on Crystal Lake (Benzie County, Michigan, USA). Within the watershed, land is primarily forested (46%), with 13% classified as rangeland, 15% designated as wetlands, 10% covered in water, and 8% used for agriculture. Urban areas cover only 8% of the watershed [30]. The Village of Beulah maintains a public water and sanitary sewer system, with sanitary waste pumped into lagoons to the south of the village. There are three stormwater outflows onto the public beach and several that discharge into Cold Creek (which is the largest tributary of Crystal Lake). The topography of the commercial street that runs parallel to the public beach directs most surface stormwater to the beach area, primarily via impervious surfaces [30]. In 2021, 11 sampling locations were chosen in collaboration with CLWA board members and Benzie Conservation District staff as areas of high interest due to previous water sampling (Fig 1). Four of the chosen sites were from small inlets around Crystal Lake, and seven were within the Cold Creek Watershed. An additional three inlet sites were added on the last day of sampling per community partner requests. In 2022, sites were chosen based on data from the previous year and where additional information was needed to clarify results. Samples were taken along Cold Creek (CC), at Beulah Beach in Crystal Lake (BB-CL), at the stormwater outflow (BB-SW), at the Crystal Avenue stormwater outflow (CAO), Shadko Creek (SC), Harris Creek (HC), Bellow’s Creek (BC), and Glen Rhoda (GR). Three sites were added in 2022 that had never been sampled before. After three sampling dates, one of those sites (CC-03+) was eliminated in favor of sampling CC-06, which was sampled in 2021, to measure contamination levels of the north/middle branch of Cold Creek before it coalesced with the south branch of Cold Creek. In total, there were 17 sites sampled throughout the two-year study.

There are three main branches of Cold Creek. Site CC-09 is the most upstream sampling site on the north branch of Cold Creek and feeds into CC-06. CC-05 + is the most upstream sampling site on the middle branch of Cold Creek, which feeds into CC-05. The north and middle branches of Cold Creek coalesce into CC-04. Thus, these sites collectively are called the north/middle branch of Cold Creek. CC-10 is on the opposite side of the road as CC-05 and feeds into the southern branch of Cold Creek. CC-03 + is the most upstream site on the south branch of Cold Creek, and it combines with CC-10 to feed into CC-03. Thus, these sites are called the south branch of Cold Creek. CC-03 and CC-04 both converge to enter a settling pond. CC-02 is at the outflow of the settling pond, and CC-01 is at the outflow of Cold Creek into Crystal Lake.

### Sample methods

From 2013–2015, 43 samples were taken by the Michigan Department of Environmental Quality (MDEQ) (currently known as Environment, Great Lakes and Energy – EGLE), at one beach on Crystal Lake (Beulah Beach) and tested for *E. coli*. Additionally, 89 samples were taken in the area between 2017 and 2020 and analyzed by SOS Analytical in Traverse City, MI, a state testing laboratory, which uses standard colony counting techniques to enumerate *E. coli* [32].

In 2021, duplicate samples were collected by a biologist from the Benzie Conservation District and a summer intern every week for seven weeks between 6/30/21 and 8/11/21 using the Method 1611 collection protocol [14]. In 2022, duplicate samples were taken every two weeks between 6/1/22 and 8/24/22 and collected identically to 2021. Samples were kept on ice and immediately transported to the laboratory at Freshwater Solutions, LLC (FWS) in Cedar, MI (<50 km from sampling sites), where they were filtered within six hours of collection. Filters were frozen at −20 °C until extraction could be completed the following day or extracted immediately. At each location sampled for each period, an additional sample was collected and sent to SOS Analytical in Traverse City, MI.. Precipitation data from the NOAA National Weather Service Beulah 7SSW, Michigan station were summed for seven days, 48 hours and 24 hours before each sampling event.

DNA extraction was performed using the Qiagen DNeasy Blood & Tissue kit per the manufacturer’s directions with a physical disruption step after adding proteinase K and buffer AL. qPCR analysis for *Enterococcus* was completed as previously reported using a modified version of the US EPA Method 1611 that compares sample cycle threshold values to a known-quantity standard curve [15]. PCR inhibition was assessed following the protocol described in US EPA Method 1611 [14]. Samples were run in duplicate on the Applied Biosystems QuantStudio 3 qPCR thermocycler. All samples were assessed for the HF183, DG3 and CG0F1-Bac markers per published protocols [18,19,33]. Assay sequences are presented in Table 1.

**Table 1:** Primer and probe sequences for the *Enterococcus*, HF183, DG3 and CG0F1-Bac qPCR assays used in this study.

Assay	Forward primer (5’-3’)	Reverse primer (5’-3’)	TaqMan Probe (5’-3’)	Reference
*Enterococcus*	GAGAAATTCCAAACGAACTTG	CAGTGCTCTACCTCCATCATT	TGGTTCTCTCCGAAATAGCTTTAGGGCTA	U.S. Environmental Protection Agency (2012a)
HF183 (human)	ATCATGAGTTCACATGTCCG	CGTAGGAGTTTGGACCGTGT	CTGAGAGGAAGGTCCCCCACATTGGA	Haugland et al. (2010)
DG3 (dog)	TTTTCAGCCCCGTTGTTTCG	TGAGCGGGCATGGTCATATT	AGTCTACGCGGGCGTACT	Green et al. (2014)
CG0F1-Bac (Canada goose)	GTAGGCCGTGTTTTAAGTCAGC	AGTTCCGCCTGCCTTGTCTA	CCGTGCCGTTATACTGAGACACTTGAG	Fremaux et al. (2010)

Technical replicates of each water sample were run during qPCR analysis. Any samples for which the technical replicates were incongruous were re-assessed for clarification and if again showed an incongruous result the two copy/reaction numbers were averaged and used to calculate GE/100 mL. No qPCR inhibition was detected in any of the samples.

### Water quality standards

While the EPA relies on individual states within the US to set water quality standards (WQS), many, including Michigan, have yet to do so for *Enterococcus* using qPCR. Michigan has developed a WQS for *E. coli* of 130 *E. coli*/100mL as the maximum acceptable level of a 30-day geometric mean. At the same time, a single-day value cannot exceed 300 *E. coli*/100 mL without eliciting action at the sampling location [34]. To compare the *E. coli* data to the *Enterococcus* qPCR, the US EPA statistical threshold value (STV) for an estimated illness rate (NGI) of 32/1000 primary contact recreators (1,280 calibrator cell equivalents/genome equivalents per 100 mL) was used [7].

### Statistical analysis

Duplicate sample data for each site on a given day were combined, and the highest *Enterococcus* value was taken for analysis. If either sample showed positive for human, dog, or Canada goose markers, they were labelled as ‘positive.’ We added 1 to all *E. coli* and *Enterococcus* data and log10 transformed them prior to analysis. For the comparison analysis between *E. coli* and *Enterococcus,* each were rated ‘1’ if they fell below the single day WQS or a ‘2’ if they fell above the single day WQS, and percent agreement was assessed for the data. Then, a Cohen’s Kappa analysis was run on these data. Additionally, a Spearman’s rank correlation coefficient (rho) test was used to analyze the relationship between log_10_ transformed *E. coli* levels (*E. coli*/100 mL) and log_10_ transformed *Enterococcus* levels (GE/100 mL) due to the non-parametric nature of these data.

To investigate the relationship between precipitation amount and *Enterococcus* levels, a Kruskal-Wallis test was run on data grouped based on level of precipitation from the previous 24 hours, 48 hours, and 7 days. Further, Dunn’s multiple comparison tests were run to compare *Enterococcus* values of each group, based on level of precipitation, to *Enterococcus* values in the 0.0 cm precipitation group. All analyses were run in R Studio (Version 2022.12.0 + 353) [35] except for the Kruskal-Wallis analysis, which was performed in GraphPad Prism 10.0.0 (Boston, Massachusetts).

## Results

Of the samples taken by the MDEQ between 2013–2015 six of the 43 samples (14%) yielded a WQS value of over 300 *E. coli*/100 mL Between 2017 and 2020, of the 89 water samples taken in the area, 27 of them (30%) were found to have *E. coli* values over the WQS of 300 *E. coli*/100 mL, while the rest of the samples had smaller though non-zero values (S1 Table). In 2021 and 2022, 33% (62 out of the 187) of *E. coli* water samples were considered contaminated enough to signal a beach posting or closure.

**Fig 2:**
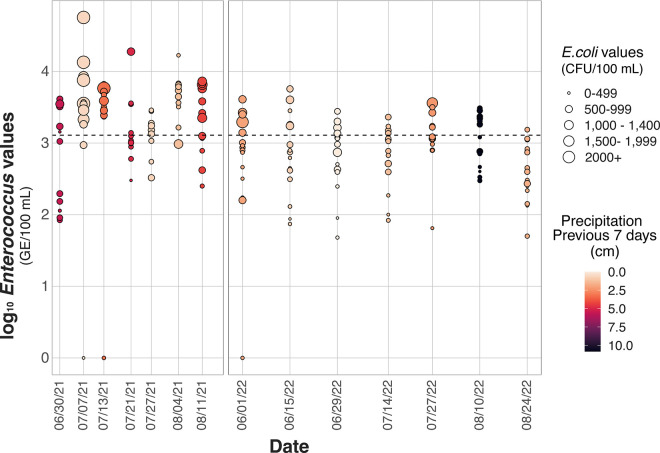
Log_10_ of *Enterococcus* values (GE/100 mL) compared to the sampling date. Coloring shows the total precipitation of the previous 7 days (cm). Size is based on *E. coli* results (CFU/100 mL). Dotted line signifies the statistical threshold value (STV) of 1,280 GE/100 mL for *Enterococcus*.

**Fig 3:**
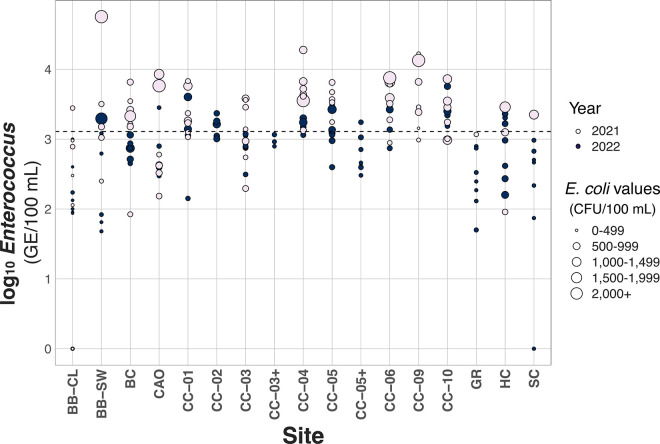
Log_10_ *Enterococcus* values (GE/100 mL) at each sampling site on Cold Creek or Crystal Lake, MI colored by year of sampling and sized based on *E. coli* results (CFU/100 mL). Dotted line signifies the statistical threshold value (STV) of 1,280 GE/100 mL for *Enterococcus*.

In 2021 and 2022, only 3 of 184 water samples returned negative for *Enterococcus*. Forty eight percent (88 out of the 184) of samples showed an *Enterococcus* level that high enough to warrant source tracking, according to the STV of 1,280 GE/100 mL (Fig 2). In 2021 there were 55 water samples over this threshold, while in 2022 only 33 water samples were above this threshold (Fig 3).

Sites CC-09, CC-04, CC-06, CC-10, CC-05 and CC-01 all had median *Enterococcus* levels over the STV of 1,280 GE/100 mL. In the north and middle branches of Cold Creek (CC-09, CC-06, CC-05, CC-05+ and CC-04), 74% of samples (39 out of 53) were over the STV. In the South branch of Cold Creek (CC-10, CC-03+ and CC-03), 55% of samples were over the STV. CC-02 (outflow of the settling pond) had 57% of samples over the STV, while CC-01 (entrance of Cold Creek to Crystal Lake) had 71% of samples over the STV (Fig 3). According to *E. coli* data, 43% of samples from the middle branch of Cold Creek were above the STV (23 out of 53), and 33% from the south branch of Cold Creek were above the STV (10 out of 30).

**Fig 4:**
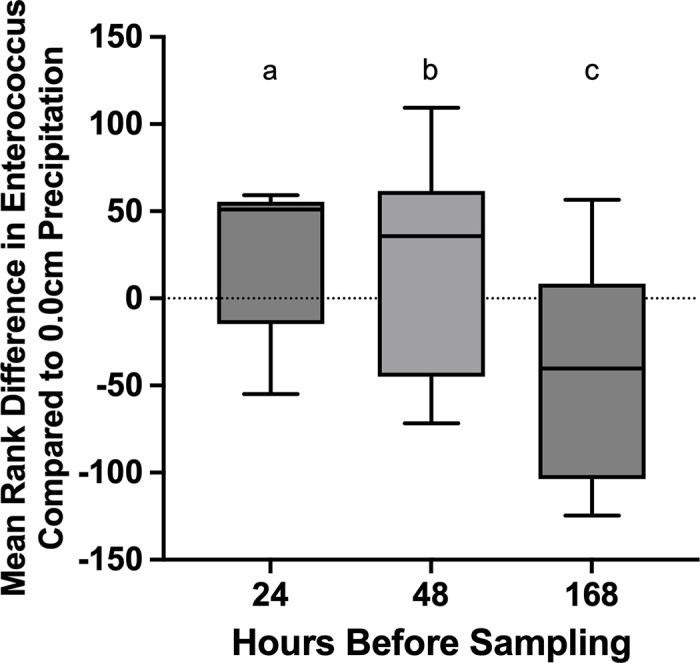
No significant differences were observed between the *Enterococcus* levels at 24, 48 and 168 hours post-rainfall and zero rainfall *Enterococcus* measurements. A Kruskal-Wallis test suggests that each time point is statistically different from the others. However, Dunn’s multiple comparison tests indicate that increased precipitation does not necessarily lead to an increase in *Enterococcus.* Letters indicate significant differences between each treatment (Kruskal-Wallis test).

*Enterococcus* levels, grouped by amount of precipitation for the previous 24 hours, 48 hours and 7 days showed a significant difference between groups, according to a Kruskal-Wallis test (Fig 4; S2 Table). When these data were further tested with a Dunn’s multiple comparison test, there was no significant relationship between higher levels of precipitation and more *Enterococcus* in water samples. When considering precipitation from the previous 24 hours, only one out of 5 precipitation levels (0.53 cm precipitation group), had *Enterococcus* levels that were significantly different than *Enterococcus* levels in the 0.0 cm precipitation group. Three precipitation levels out of 11 (0.13 cm, 0.53 cm, and 1.07 cm groups) had *Enterococcus* levels that were significantly different from the 0.0 cm precipitation group when measuring the previous 48 hours of precipitation. Similarly, when measuring precipitation for the previous 7 days, four out of 13 groups (0.58 cm, 1.24 cm, 3.28 cm, and 4.24 cm) had *Enterococcus* levels significantly different than those of the 0.0 cm precipitation group. In all tests, there was no scientifically plausible explanation for the sporadic groups that had a significant relationship. This type of analysis could be impacted by the fact that this data is not homoscedastic, so additional analyses may be needed to further investigate the relationship between *Enterococcus* values and precipitation. The lowest precipitation day showed some of the highest *Enterococcus* values of the study period (Fig 3).

**Fig 5:**
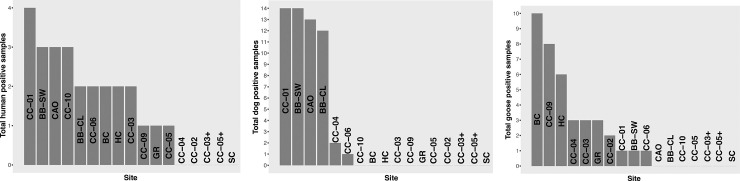
Number of samples that were positive for HF183 (human), Dog3 (dog), and CG0F1-Bac (Canada goose) at each sampling site. Note the different scales on the y-axis of each graph.

Of the 184 samples analyzed for *Enterococcus*, 26 were found positive for HF183. Of these positive samples, 15 were found at outflows into Crystal Lake, near the public beach. One site had four positive HF183 samples (CC-01), while three sites had three HF183 positive samples (BB-SW, CAO, and CC-10). Five sites had no positives for the marker (CC-02, CC-03 + , CC-04, CC-05 + , SC) (Fig 5). Notably, there are stormwater outflows upstream of CC-01 and at BB-SW and CAO, which had three of the four highest numbers of HF183 positive samples.

Fifty-six samples tested positive for dog fecal contamination, with CC-01, BB-SW, CAO and BB-CL having all but three of the positive results. Ten sites tested positive for Canada goose contamination, with a total of 38 positive samples. The three highest sites for Canada goose contamination were BC, CC-09 and HC (10, 8 and 6 positive samples, respectively).

The north/middle branch of Cold Creek had 8% of samples (4 of 53) positive for human fecal contamination, while the south branch of Cold Creek had 16% (5 of 31) positive for HF183. CC-02 (water leaving the settling pond) did not possess human fecal contamination in any of the samples, but CC-01 (inflow to Crystal Lake) showed 29% of samples (4 of 14) positive. Of the 17 sampling locations, 12 showed at least some amount of human fecal contamination.

**Fig 6:**
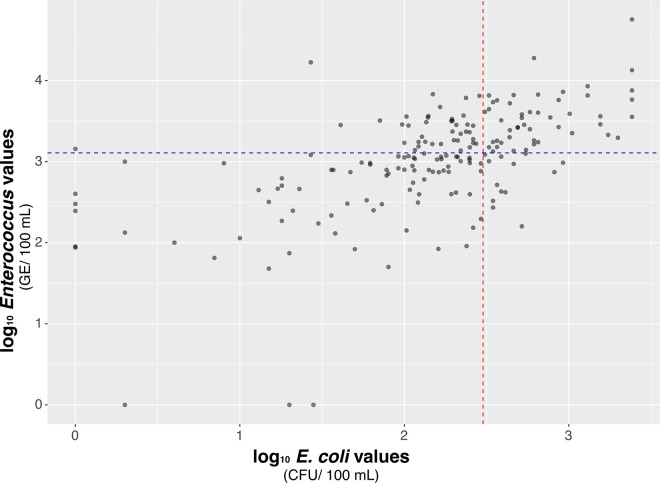
Comparison of log_10_ *Enterococcus* values (GE/100 mL) as measured with qPCR to log_10_ *E. coli* values (CFU/100 mL) as measured with culture-based methods. The blue line represents the standard threshold value (STV) for *Enterococcus* of 1,280 GE/100 mL. The red line represents the single-day water quality standard (WQS) of 300 CFU/100 mL for *E. coli.*

In the percent agreement analysis comparing *Enterococcus* qPCR and *E. coli* culture data, 71% of samples were in agreement on whether the sample value would have yielded a beach management decision. Of the samples tested, 26% were above the single day WQS for both *E. coli* and *Enterococcus*, and 45% were below the single day WQS for both FIB (Fig 6). A Cohen’s Kappa test of these data showed an unweighted kappa = 0.42, which implies a moderate agreement between these two variables [36]. Additionally, the Spearman’s rho analysis indicated a positive association between the *E. coli* and *Enterococcus* data (rho = 0.608, p < 0.001).

## Discussion

Our study demonstrates the value of CBM to address monitoring and research questions that interest scientists and local community members. This community-based study provided insight into enteric bacteria levels at a historically contaminated area (S3 Table) and clarified the source of that contamination as being partially of human origin. Human, dog, and Canada goose contamination were all assessed and found in water samples during our study. However, when considering results from a risk perspective, we focus on the 26 samples that were positive for human contamination across 12 sampling sites due to the increased chance of human fecal contamination carrying other human disease-causing agents [7].

This study allowed us to compare two different FIB and use MST to assess the origin of the fecal contamination. We observed a moderate positive relationship between values when comparing culture-based *E. coli* monitoring methods to qPCR-based *Enterococcus* monitoring methods. This is unsurprising as past research has focused on the comparison of these two species as FIB, revealing a low correlation between the results of their respective tests (R = 0.60 and 0.69 in two different testing methods) [37]. *Enterococcus* is thought to persist longer in the environment, thus making it a more conservative indicator [38]. It has also been shown that qPCR results do not closely correlate with culture-based methods even when measuring the same species, likely due to the fact that qPCR-based assessment will detect live (culturable), viable but not culturable (VBNC) and dead bacteria [12].

Standard *E. coli* testing protocols limit the ability to source track positive samples, leaving in question the source of this fecal contamination. If only *E. coli* culture data and *Enterococcus* qPCR data had been collected from the two branches of Cold Creek, we would have concluded that the north/ middle branch of Cold Creek displayed greater FIB contamination. We would have missed that the south branch of Cold Creek had a higher persistence of HF183 contamination. This would have left out a significant aspect of our conclusions and left uncertainty with respect to the source of the pollution in our results. For this study, being able to source track for human, dog and goose fecal contamination was vital to meeting the needs of community partners, especially considering that human fecal contamination of recreational water may be linked to more GI illnesses than contamination from certain other sources (i.e., gull) [39] qPCR results do overestimate the number of times a beach action would need to be taken when compared to *E. coli* data, perhaps due to DNA detection from non-viable *Enterococcus*. However, just because *Enterococcus* is not culturable does not mean it did not come from a source that could reflect a risk for GI illness [12]. Thus, results should be carefully considered when examining culture-based *E. coli* testing alone.

Precipitation in the 24 hours, 48 hours and seven days preceding sample collection was assessed to determine whether precipitation influenced FIB, HF183, DG3 and CG0F1-Bac presence and abundance. Previous studies have demonstrated a relationship between FIB and precipitation [40–45]. Precipitation measured over the previous 24 hours, 48 hours, or seven days did show a significant association with *Enterococcus* levels grouped by amount of precipitation, measured by a Kruskal-Wallis test, however a Dunn’s multiple comparison test found no significant relationship between high precipitation levels and more bacteria in the water. Precipitation did not relate to HF183, dog or Canada goose positive samples. We conclude that the observed increases in bacterial levels in the water were not due to runoff events.

*Enterococcus* levels varied in specific branches of Cold Creek. CC-03 and CC-04 are located at the inflow to a settling pond, put in place to decrease the sediment that ultimately makes its way into Crystal Lake. CC-02 is a sampling location added in 2022 to help clarify the settling pond’s relationship with bacterial levels. CC-04 is the entry point of the north branch of Cold Creek into the settling pond and shows consistently high values for *Enterococcus* but no detectable contamination from human fecal material. CC-09, a part of the north branch of Cold Creek, has historically had a lot of goose activity and eight positive samples for goose fecal contamination. Thus, we can conclude that geese are likely a contributing source to the high *Enterococcus* values at this site. CC-03 is the entry point of the south branch of Cold Creek into the settling pond and shows much lower levels of *Enterococcus* than CC-04 but does show human fecal contamination. On the other side of the settling pond, CC-02 shows *Enterococcus* results to be a bit higher than CC-03 in 2022 but lower than CC-04, perhaps showing a mix of highly contaminated water with low-contaminated water. Of note is the sanitary waste lagoon with spray irrigation wastewater treatment system which receives sanitary waste from the Village of Beulah and is located southwest of our sampling sites. This system, which includes six lagoons and 12 spray irrigation zones, has been deemed ‘failing’ and work is approved to upgrade the wastewater treatment plant [46]. This failing system could contribute fecal material to the groundwater in our study, however it most likely feeds into a river downstream of Crystal Lake. The relationship is unknown at this time.

Human fecal pollution was more frequently observed in the south branch of Cold Creek than the north/middle branch of Cold Creek. Future research should focus on this area to better specify the source of this contamination. Interestingly, no human fecal material was found at the outflow of the settling pond. There has been conflicting conclusions about the effect of UV light on enteric bacteria measured by qPCR, with some studies showing no effect of UV light on *Enterococcus* levels [47,48] or HF183 levels [49,50] as measured by qPCR, while others have shown sunlight to significantly decrease enteric bacteria levels [51]. Culturable bacteria became unmeasurable much sooner than those measured by qPCR, suggesting that the DNA of dead bacteria may persist in the environment after viable bacteria have died [50]. Perhaps the HF183 marker decayed due to prolonged exposure to UV radiation while in the settling pond, so none was found at the outflow.

The use of HF183 as an MST indicator has had mixed results regarding the cross-reactivity with dog fecal contamination. One study showed no cross-reactivity [16], while others have shown some cross-reactivity [52–54]. All water samples from sites CC-01, BB-SW, and CAO that were positive for HF183 were also positive for DG3. Because of the potential for cross-reactivity between the HF183 assay and dog feces, we cannot conclusively say the fecal contamination is of human origin. However, none of the water samples taken from sites BC, CC-03, CC-05, CC-09, CC-10, GR and HC that were positive for HF813 contamination were positive for DG3 contamination, which suggests that there is human fecal contamination in our study area. Site CC-06 had two water samples positive for HF183, but only one of them was also positive for DG3. BB-CL also showed high dog fecal contamination and had two positive HF183 samples. CC-01, BB-SW, CAO and BB-CL are in areas of public use with many impervious surfaces while also being the sites most greatly impacted by stormwater outflows.

Dominant sources of fecal contributions in a single area have been shown to vary depending on the time of year and location within a watershed [23,55]. Thus, the presentation of dog, Canada goose, and human fecal pollution could have naturally varied over the sampling period. Due to this possibility and potential cross-reactivity with dog markers, it may be recommended to include caffeine sampling or another confirmatory test for human contamination [22].

Along with the outflow into Crystal Lake, the south branch of Cold Creek (specifically CC-10) should be examined for HF183 sources, as it had a consistent HF183 signal throughout the study and has low potential for cross-reactivity with the dog MST marker. BC, CC-09 and HC were the sites most greatly impacted by goose fecal contamination (6 or more positive samples). BC drains a public park that may be appropriate habitat for geese, while HC is a wooded area that drains some orchards at the head of the watershed. CC-09 is near a wetland area, with a pond upstream that could house geese during certain times of the year.

One drawback of using HF183 as an MST target is the low persistence in the environment compared to FIB [50,56]. The low number of samples found to be positive can be challenging to analyze. This was found in one study examining sanitation issues in central Appalachia [57]. It was suggested that a general FIB should also be sampled along with HF183 to help clarify results. The HF183 marker gene is also known to decay faster in the environment than pathogen genes do [58]. Thus, the absence of HF183 markers in the water does not mean there is zero risk of infectious agents in water. Because of this, we suggest that any site with even one positive sample for HF183 found there would be considered to have human fecal contribution.

When considering recreational swimming, many factors may impact an individual’s risk of developing gastrointestinal illness (GI). While this was not a focus of the study since many of our sample locations are not recreational swim locations, public interest in health risks are often pertinent. The most common pathogen shown to persist in contaminated water is norovirus, which can persist in ambient waters for up to 61 days [59]. This is much longer than the average persistence of HF183 [50], meaning that if there is a contribution of human fecal material into a water source, there may be a risk of gastrointestinal illness many days after the HF183 marker is found [60]. While none of the sampling sites along Cold Creek have recreational use (including the inflow to Crystal Lake), the sampling site at Beulah Beach (BB-CL) has many swimmers throughout the summer. Interestingly, the Beulah Beach samples possessed significantly lower *Enterococcus* values than the Cold Creek samples but did have two HF183-positive samples. This is the only site in our study where detectable human fecal contamination was present in water in direct contact with swimmers, which may simultaneously be the source of human fecal contamination for that site.

Stormwater outflows and septic systems may more likely contribute to human fecal material at the sites without recreational swimming. Septic system density has been linked to increased fecal contamination [61] in the water, specifically from human sources [29,62]. Stormwater systems have also been linked to high FIB and human MST numbers [21,23–28]. We noticed that the highest *Enterococcus* levels were found on a day without rain the previous seven days, not linking those bacterial levels to runoff due to precipitation. One study showed no link between precipitation and human fecal bacteria in stormwater outflows [25], which is consistent with our findings. We hypothesize that these high values were due to point source contamination that consistently enters the system but is generally diluted with greater precipitation, especially since stormwater outflows in our study have continuous flow, not just during rain events. During low precipitation times, the bacteria would coalesce in higher levels in the water.

As a community, evidence from this study shows the need to further investigate bacterial contamination sources between the settling pond and outflow into Crystal Lake. *Enterococcus* was at lower levels when leaving the settling pond, with no HF183 found, than when it entered Crystal Lake. Thus, the water gained *Enterococcus*, human and dog fecal contamination between leaving the settling pond and getting to Crystal Lake. Three stormwater outflows between the settling pond and the sampling site entering Crystal Lake are likely sources of contamination. The stormwater sewer system for the Village of Beulah is currently unmapped and mapping this system while checking the integrity of infrastructure would benefit the community to further clarify where the HF183 and DG3 influence may be coming from. While the Village of Beulah is on a sewer system, several houses have individual septic systems that are not tied into the sewer system and could be contamination sources, although these are a less likely source.

While the use of qPCR has dramatically increased specificity of microbial water testing and allowed flexibility in the timing of sampling, extraction and analysis, there are still limitations in what target sequences are looked for and the amount of information provided from those specific targets. An expanded target list may give further clarification on contamination source(s). However, the chosen targets were informed by local knowledge provided by CBM partners as the most probable causes of contamination. Using newer technology to test environmental DNA (eDNA) along with metabarcoding has shown great promise for the future of microbial and invasive species water testing [63–65]. This testing would allow a greater understanding of the bacterial community and its dynamics within a watershed. The ability to test for all bacterial species in a water sample would be beneficial as we would not be limited to a specific target. With this methodology, we may see species emerge as influential to water quality that have yet to be a focus of testing via DNA-based water monitoring. eDNA testing could improve microbial water testing for communities such as the one in this study, which are looking for source points of contamination and hoping to provide data to compel change in their community.

Although community-based monitoring is an effective way to collect numerous water samples over a short period [4], studies have yet to be published in which this framework focuses on enteric bacteria monitoring to answer citizen questions. This community-based study showed results that correspond to current literature about contamination of water in municipalities while collecting a plethora of samples with the help of community partners. Specifically, this study provided meaningful information for community members about the water quality of Cold Creek, while exemplifying the benefit of using qPCR to shed light on the source of contamination in historically contaminated waters.

## Supporting information

S1 Table Site GPS coordinates for Crystal Lake and Cold Creek. (DOCX)

S2 Table Results from Kruskal-Wallis test with Dunn’s multiple comparison test for *Enterococcus* values grouped by precipitation amount. (DOCX)

S3 Table Historical (2017–2020) *E. coli* testing data from locations near Crystal Lake, MI, with 2021 and 2022 data. (DOCX)

## References

[1] Conrad CT, Daoust T. Community-based monitoring frameworks: increasing the effectiveness of environmental stewardship. Environ Manage. 2008;41(3):358–66. doi: 10.1007/s00267-007-9042-x 18026783

[2] Dickinson JL, Shirk J, Bonter D, Bonney R, Crain RL, Martin J, et al. The current state of citizen science as a tool for ecological research and public engagement. Frontiers in Ecol & Environ. 2012;10(6):291–7. doi: 10.1890/110236

[3] Robinson CV, Baird DJ, Wright MTG, Porter TM, Hartwig K, Hendriks E. Combining DNA and people power for healthy rivers: Implementing the STREAM community-based approach for global freshwater monitoring. Perspect Ecol Conserv. 2021;19(3):279–85.

[4] Rudko SP, Reimink RL, Peter B, White J, Hanington PC. Democratizing water monitoring: Implementation of a community-based qPCR monitoring program for recreational water hazards. PLoS One. 2020;15(5):e0229701. doi: 10.1371/journal.pone.0229701 32401786 PMC7219769

[5] Storey RG, Wright-Stow A. Community-based monitoring of New Zealand stream macroinvertebrates: agreement between volunteer and professional assessments and performance of volunteer indices. N Z J Mar Freshw Res. 2017;51(1):60–77.

[6] Fewtrell L, Bartram J. Water quality: guidelines, standards, and health: assessment of risk and risk management for water-related infectious disease. Geneva: World Health Organization. 2001.

[7] Department of Environmental Quality. Recreational Water Quality Criteria. 2012. https://www.epa.gov/sites/default/files/2015-10/documents/rwqc2012.pdf

[8] EPA Region 4 (Science and Ecosystem Support Division). Approval of Colilert-18 for the Detection and Enumeration of Fecal Coliforms in Wastewater Samples. 2010. https://www.epa.gov/sites/default/files/2015-06/documents/r4colilert-18.pdf

[9] Noble RT, Blackwood AD, Griffith JF, McGee CD, Weisberg SB. Comparison of rapid quantitative PCR-based and conventional culture-based methods for enumeration of *Enterococcus* spp. and *Escherichia coli* in recreational waters. Applied and Environmental Microbiology. 2010;76(22):7437–43. doi: 10.1128/AEM.01368-10 20870786 PMC2976187

[10] Lane MJ, McNair JN, Rediske RR, Briggs S, Sivaganesan M, Haugland R. Simplified Analysis of Measurement Data from A Rapid E. coli qPCR Method (EPA Draft Method C) Using A Standardized Excel Workbook. Water (Basel). 2020;12(3):1–775. doi: 10.3390/w12030775 32461809 PMC7252523

[11] Gonzalez RA, Noble RT. Comparisons of statistical models to predict fecal indicator bacteria concentrations enumerated by qPCR- and culture-based methods. Water Res. 2014;48:296–305. doi: 10.1016/j.watres.2013.09.038 24139103

[12] Wade TJ, Calderon RL, Brenner KP, Sams E, Beach M, Haugland R, et al. High sensitivity of children to swimming-associated gastrointestinal illness: results using a rapid assay of recreational water quality. Epidemiology. 2008;19(3):375–83. doi: 10.1097/EDE.0b013e318169cc87 18379427

[13] Wade TJ, Arnold BF, Schiff K, Colford JM Jr, Weisberg SB, Griffith JF, et al. Health risks to children from exposure to fecally-contaminated recreational water. PLoS One. 2022;17(4):e0266749. doi: 10.1371/journal.pone.0266749 35413082 PMC9004770

[14] U.S. Environmental Protection Agency. Method 1611: Enterococci in Water by TaqMan Quantitative Polymerase Chain Reaction (qPCR) Assay. Washington, D.C.: U.S. Environmental Protection Agency. 2012. https://www.epa.gov/sites/default/files/2015-08/documents/method_1611_2012.pdf

[15] Rudko S. Development and implementation of a community based qPCR monitoring program for biological hazards of recreational water. 2020. https://era.library.ualberta.ca/items/4e3bf6b2-67eb-420d-9419-171a3cbce949 10.1371/journal.pone.0229701 PMC7219769 32401786

[16] Ahmed W, Stewart J, Powell D, Gardner T. Evaluation of Bacteroides markers for the detection of human faecal pollution: Evaluation of the host-specific Bacteroides marker. Lett Appl Microbiol. 2007;46(2):237–42. 18028325 10.1111/j.1472-765X.2007.02287.x

[17] Johnston C, Byappanahalli MN, Gibson JM, Ufnar JA, Whitman RL, Stewart JR. Probabilistic analysis showing that a combination of *Bacteroides* and *Methanobrevibacter* source tracking markers is effective for identifying waters contaminated by human fecal pollution. Environ Sci Technol. 2013;47(23):13621–8. doi: 10.1021/es403753k 24182330

[18] Fremaux B, Boa T, Yost CK. Quantitative real-time PCR assays for sensitive detection of Canada goose-specific fecal pollution in water sources. Applied and Environmental Microbiology. 2010;76(14):4886–9. 20511425 10.1128/AEM.00110-10 PMC2901712

[19] Green HC, White KM, Kelty CA, Shanks OC. Development of rapid canine fecal source identification PCR-based assays. Environmental Science & Technology. 2014;48(19):11453–61. 25203917 10.1021/es502637b

[20] Brownell MJ, Harwood VJ, Kurz RC, McQuaig SM, Lukasik J, Scott TM. Confirmation of putative stormwater impact on water quality at a Florida beach by microbial source tracking methods and structure of indicator organism populations. Water Res. 2007;41(16):3747–57. doi: 10.1016/j.watres.2007.04.001 17544051

[21] Petersen TM, Rifai HS, Suarez MP, Stein AR. Bacteria loads from point and nonpoint sources in an urban watershed. J Environ Eng. 2005;131(10):1414–25.

[22] Sidhu JPS, Ahmed W, Gernjak W, Aryal R, McCarthy D, Palmer A. Sewage pollution in urban stormwater runoff as evident from the widespread presence of multiple microbial and chemical source tracking markers. Sci Total Environ. 2013;463–464:488–96. 10.1016/j.scitotenv.2013.06.020 23831795

[23] Stein ED, Ackerman D. Dry Weather Water Quality Loadings in Arid, Urban Watersheds of the Los Angeles Basin, California, USA. J Am Water Resour Assoc. 2007;43(2):398–413.

[24] Ahmed W, Hamilton K, Toze S, Cook S, Page D. A review on microbial contaminants in stormwater runoff and outfalls: Potential health risks and mitigation strategies. Sci Total Environ. 2019;692:1304–21. doi: 10.1016/j.scitotenv.2019.07.055 31539962 PMC7126443

[25] Sauer EP, Vandewalle JL, Bootsma MJ, McLellan SL. Detection of the human specific Bacteroides genetic marker provides evidence of widespread sewage contamination of stormwater in the urban environment. Water Res. 2011;45(14):4081–91. doi: 10.1016/j.watres.2011.04.049 21689838

[26] Sercu B, Van De Werfhorst LC, Murray J, Holden PA. Storm drains are sources of human fecal pollution during dry weather in three urban southern California watersheds. Environ Sci Technol. 2009;43(2):293–8. doi: 10.1021/es801505p 19238954

[27] Sidhu JPS, Hodgers L, Ahmed W, Chong MN, Toze S. Prevalence of human pathogens and indicators in stormwater runoff in Brisbane, Australia. Water Res. 2012;46(20):6652–60. doi: 10.1016/j.watres.2012.03.012 22572123

[28] Staley ZR, Grabuski J, Sverko E, Edge TA. Comparison of microbial and chemical source tracking markers to identify fecal contamination sources in the Humber River (Toronto, Ontario, Canada) and associated storm water outfalls. Applied and Environmental Microbiology. 2016;82(21):6357–66. 27542934 10.1128/AEM.01675-16 PMC5066352

[29] Verhougstraete MP, Martin SL, Kendall AD, Hyndman DW, Rose JB. Linking fecal bacteria in rivers to landscape, geochemical, and hydrologic factors and sources at the basin scale. Proc Natl Acad Sci U S A. 2015;112(33):10419–24. doi: 10.1073/pnas.1415836112 26240328 PMC4547304

[30] McCauley M, Gest S, Hoogterp E. Betsie River/ Crystal Lake Watershed Management Plan. 2016. https://www.networksnorthwest.org/userfiles/filemanager/1dncmmwlq9y5w6hsswc8/

[31] Michigan Department of Environment, Great Lakes, and Energy. Beach Guard: Crystal Lake- Beulah Beach. 2022. https://www.egle.state.mi.us/beach/BeachDetail.aspx?BeachID=3718

[32] U.S. Environmental Protection Agency. Method 1603: Excherichia coli (E. coli) in water by membrane filtration using modified membrane-thermotolerant Escherichia coli agar (modified mTEC). 2002. https://nepis.epa.gov/Exe/ZyNET.exe/P1008MZV.txt?ZyActionD=ZyDocument&Client=EPA&Index=2000%20Thru%202005&Docs=&Query=&Time=&EndTime=&SearchMethod=1&TocRestrict=n&Toc=&TocEntry=&QField=&QFieldYear=&QFieldMonth=&QFieldDay=&UseQField=&IntQFieldOp=0&ExtQFieldOp=0&XmlQuery=&File=D%3A%5CZYFILES%5CINDEX%20DATA%5C00THRU05%5CTXT%5C00000025%5CP1008MZV.txt&User=ANONYMOUS&Password=anonymous&SortMethod=h%7C-&MaximumDocuments=1&FuzzyDegree=0&ImageQuality=r75g8/r75g8/x150y150g16/i425&Display=hpfr&DefSeekPage=x&SearchBack=ZyActionL&Back=ZyActionS&BackDesc=Results%20page&MaximumPages=1&ZyEntry=1

[33] Haugland RA, Varma M, Sivaganesan M, Kelty C, Peed L, Shanks OC. Evaluation of genetic markers from the 16S rRNA gene V2 region for use in quantitative detection of selected Bacteroidales species and human fecal waste by qPCR. Syst Appl Microbiol. 2010;33(6):348–57. doi: 10.1016/j.syapm.2010.06.001 20655680

[34] Department of Environmental Quality. Water Quality Standards. 2006. https://www.michigan.gov/egle/-/media/Project/Websites/egle/Documents/Programs/WRD/NPDES/part-4-water-quality-standards.pdf?rev=e373af3ede7d4036a2da5159b63db7f2&hash=DC11FB2DE2138522CB23B233E08B8177

[35] R Core Team. A language and environment for statistical computing. 2023. https://www.R-project.org/

[36] Landis JR, Koch GG. The measurement of observer agreement for categorical data. Biometrics. 1977;33(1):159–74. 843571

[37] Kinzelman J, Ng C, Jackson E, Gradus S, Bagley R. Enterococci as indicators of Lake Michigan recreational water quality: comparison of two methodologies and their impacts on public health regulatory events. Appl Environ Microbiol. 2003;69(1):92–6. doi: 10.1128/AEM.69.1.92-96.2003 12513981 PMC152387

[38] Jin G, Jeng H-W, Bradford H, Englande AJ. Comparison of *E. coli*, enterococci, and fecal coliform as indicators for brackish water quality assessment. Water Environ Res. 2004;76(3):245–55. doi: 10.2175/106143004x141807 15338696

[39] Soller JA, Schoen ME, Bartrand T, Ravenscroft JE, Ashbolt NJ. Estimated human health risks from exposure to recreational waters impacted by human and non-human sources of faecal contamination. Water Res. 2010;44(16):4674–91. doi: 10.1016/j.watres.2010.06.049 20656314

[40] Celico F, Varcamonti M, Guida M, Naclerio G. Influence of precipitation and soil on transport of fecal enterococci in fractured limestone aquifers. Appl Environ Microbiol. 2004;70(5):2843–7. doi: 10.1128/AEM.70.5.2843-2847.2004 15128541 PMC404436

[41] Islam MMM, Hofstra N, Islam MdA. The Impact of Environmental Variables on Faecal Indicator Bacteria in the Betna River Basin, Bangladesh. Environ Process. 2017;4(2):319–32. doi: 10.1007/s40710-017-0239-6

[42] Laureano-Rosario AE, Symonds EM, Rueda-Roa D, Otis D, Muller-Karger FE. Environmental Factors Correlated with Culturable Enterococci Concentrations in Tropical Recreational Waters: A Case Study in Escambron Beach, San Juan, Puerto Rico. Int J Environ Res Public Health. 2017;14(12):1602. doi: 10.3390/ijerph14121602 29257092 PMC5751019

[43] McKee BA, Molina M, Cyterski M, Couch A. Microbial source tracking (MST) in Chattahoochee River National Recreation Area: Seasonal and precipitation trends in MST marker concentrations, and associations with E. coli levels, pathogenic marker presence, and land use. Water Res. 2020;171:115435. 31927096 10.1016/j.watres.2019.115435 PMC8188702

[44] Noble RT, Weisberg SB, Leecaster MK, McGee CD, Dorsey JH, Vainik P, et al. Storm effects on regional beach water quality along the southern California shoreline. J Water Health. 2003;1(1):23–31. 15384270

[45] Walters SP, Thebo AL, Boehm AB. Impact of urbanization and agriculture on the occurrence of bacterial pathogens and stx genes in coastal waterbodies of central California. Water Res. 2011;45(4):1752–62. doi: 10.1016/j.watres.2010.11.032 21168181

[46] Michigan Department of Environment, Great Lakes, and Energy. Amendment to substantial public health risk project grant agreement between the Michigan Department of Environment, Great Lakes, and Energy and the Village of Beulah. 2022. https://www.villageofbeulah.net/_files/ugd/e1b4cc_faa978c2e9194754aadbfcbb6cf016f5.pdf

[47] Chern EC, Brenner K, Wymer L, Haugland RA. Influence of wastewater disinfection on densities of culturable fecal indicator bacteria and genetic markers. J Water Health. 2014;12(3):410–7. doi: 10.2166/wh.2013.179 25252344

[48] Dick LK, Stelzer EA, Bertke EE, Fong DL, Stoeckel DM. Relative decay of *Bacteroidales* microbial source tracking markers and cultivated *Escherichia coli* in freshwater microcosms. Applied and Environmental Microbiology. 2010;76(10):3255–62. doi: 10.1128/AEM.02945-09 20348289 PMC2869114

[49] Korajkic A, McMinn BR, Shanks OC, Sivaganesan M, Fout GS, Ashbolt NJ. Biotic interactions and sunlight affect persistence of fecal indicator bacteria and microbial source tracking genetic markers in the upper Mississippi river. Appl Environ Microbiol. 2014;80(13):3952–61. doi: 10.1128/AEM.00388-14 24747902 PMC4054226

[50] Walters SP, Field KG. Survival and persistence of human and ruminant-specific faecal *Bacteroidales* in freshwater microcosms. Environ Microbiol. 2009;11(6):1410–21. doi: 10.1111/j.1462-2920.2009.01868.x 19397677

[51] Walters SP, Yamahara KM, Boehm AB. Persistence of nucleic acid markers of health-relevant organisms in seawater microcosms: implications for their use in assessing risk in recreational waters. Water Res. 2009;43(19):4929–39. doi: 10.1016/j.watres.2009.05.047 19616273

[52] Ahmed W, Masters N, Toze S. Consistency in the host specificity and host sensitivity of the Bacteroides HF183 marker for sewage pollution tracking. Lett Appl Microbiol. 2012;55(4):283–9. doi: 10.1111/j.1472-765X.2012.03291.x 22809116

[53] Kildare BJ, Leutenegger CM, McSwain BS, Bambic DG, Rajal VB, Wuertz S. 16S rRNA-based assays for quantitative detection of universal, human-, cow-, and dog-specific fecal Bacteroidales: a Bayesian approach. Water Res. 2007;41(16):3701–15. doi: 10.1016/j.watres.2007.06.037 17644149

[54] McQuaig SM, Scott TM, Lukasik JO, Paul JH, Harwood VJ. Quantification of human polyomaviruses JC Virus and BK Virus by TaqMan quantitative PCR and comparison to other water quality indicators in water and fecal samples. Appl Environ Microbiol. 2009;75(11):3379–88. doi: 10.1128/AEM.02302-08 19346361 PMC2687276

[55] Whitlock JE, Jones DT, Harwood VJ. Identification of the sources of fecal coliforms in an urban watershed using antibiotic resistance analysis. Water Res. 2002;36(17):4273–82. doi: 10.1016/s0043-1354(02)00139-2 12420932

[56] Liang Z, He Z, Zhou X, Powell CA, Yang Y, Roberts MG, et al. High diversity and differential persistence of fecal Bacteroidales population spiked into freshwater microcosm. Water Res. 2012;46(1):247–57. doi: 10.1016/j.watres.2011.11.004 22100053

[57] Cantor J, Krometis L-A, Sarver E, Cook N, Badgley B. Tracking the downstream impacts of inadequate sanitation in central Appalachia. J Water Health. 2017;15(4):580–90. doi: 10.2166/wh.2017.005 28771155

[58] Ahmed W, Toze S, Veal C, Fisher P, Zhang Q, Zhu Z, et al. Comparative decay of culturable faecal indicator bacteria, microbial source tracking marker genes, and enteric pathogens in laboratory microcosms that mimic a sub-tropical environment. Sci Total Environ. 2021;751:141475. doi: 10.1016/j.scitotenv.2020.141475 32890804

[59] Seitz SR, Leon JS, Schwab KJ, Lyon GM, Dowd M, McDaniels M. Norovirus infectivity in humans and persistence in water. Applied and Environmental Microbiology. 2011;77(19):6884–8. doi: 10.1128/AEM.00589-11 21856841 PMC3187119

[60] Boehm AB, Soller JA, Shanks OC. Human-associated fecal quantitative polymerase chain reaction measurements and simulated risk of gastrointestinal illness in recreational waters contaminated with raw sewage. Environ Sci Technol Lett. 2015;2(10):270–5.

[61] Sowah R, Zhang H, Radcliffe D, Bauske E, Habteselassie MY. Evaluating the influence of septic systems and watershed characteristics on stream faecal pollution in suburban watersheds in Georgia, USA. J Appl Microbiol. 2014;117(5):1500–12. doi: 10.1111/jam.12614 25074241

[62] Sowah RA, Habteselassie MY, Radcliffe DE, Bauske E, Risse M. Isolating the impact of septic systems on fecal pollution in streams of suburban watersheds in Georgia, United States. Water Res. 2017;108:330–8. doi: 10.1016/j.watres.2016.11.007 27847149

[63] Garlapati D, Kumar BC, Muthukumar C, Madeswaran P, Ramu K, Murthy MVR. Assessing the in situ bacterial diversity and composition at anthropogenically active sites using the environmental DNA (eDNA). Mar Pollut Bull. 2021;170:112593. doi: 10.1016/j.marpolbul.2021.112593 34126444

[64] Nevers MB, Byappanahalli MN, Morris CC, Shively D, Przybyla-Kelly K, Spoljaric AM, et al. Environmental DNA (eDNA): A tool for quantifying the abundant but elusive round goby (Neogobius melanostomus). PLoS One. 2018;13(1):e0191720. doi: 10.1371/journal.pone.0191720 29357382 PMC5777661

[65] Wu Y, Colborne SF, Charron MR, Heath DD. Development and validation of targeted environmental DNA (eDNA) metabarcoding for early detection of 69 invasive fishes and aquatic invertebrates. Environ DNA. 2022;3(edn3.359).

