# Supplementary material for: Assessing fecal pollution source in a Northern Michigan Lake using qPCR and a community-based monitoring framework
Source: PLoS One. 2025 Aug 29;20(8):e0331494. doi: 10.1371/journal.pone.0331494 (PMC12396685; doi:10.1371/journal.pone.0331494)
Supplement: S1 Table — (DOCX) [file pone.0331494.s001.docx]

**Supplementary Table 1: Site GPS coordinates for Crystal Lake and Cold Creek.**

| **Site** | **Latitude** | **Longitude** |
| --- | --- | --- |
| BB-CL | 44.629 | -86.097 |
| BB-SW | 44.62846 | -86.097535 |
| BC | 44.660889 | -86.232194 |
| CAO | 44.627306 | -86.10075 |
| CC-01 | 44.629361 | -86.095861 |
| CC-02 | 44.6291733 | -86.093534 |
| CC-03 | 44.630167 | -86.092056 |
| CC-03+ | 44.626506 | -86.088372 |
| CC-04 | 44.630573 | -86.09209 |
| CC-05 | 44.632111 | -86.089694 |
| CC-05+ | 44.632222 | -86.078834 |
| CC-06 | 44.63219 | -86.08986 |
| CC-09 | 44.637 | -86.087 |
| CC-10 | 44.632 | -86.089 |
| GR | 44.67125 | -86.156389 |
| HC | 44.65075 | -86.101778 |
| SC | 44.645056 | -86.090028 |
| Note: CC stands for Cold Creek. CAO is the Crystal Avenue Stormwater Outflow. BB-CL is on Beulah Beach in Crystal Lake. BB-SW is the Stormwater Outflow at Beulah Beach. SC, HC, and BC were all small creek inlets, which are Shadko Creek, Harris Creek, and Bellow’s Creek, respectively. The label GR represents the Glen Rhoda tributary. | | |
