# Supplementary material for: Assessing fecal pollution source in a Northern Michigan Lake using qPCR and a community-based monitoring framework
Source: PLoS One. 2025 Aug 29;20(8):e0331494. doi: 10.1371/journal.pone.0331494 (PMC12396685; doi:10.1371/journal.pone.0331494)
Supplement: S3 Table — (DOCX) [file pone.0331494.s003.docx]

##### **S3 Table: Historical (2017-2020) *E. coli* testing data from locations near Crystal Lake, MI, with 2021 and 2022 data.**

| **Date** | **CC-01** | **CAO** | **CC-03** | **CC-04** | **CC-05** | **CC-06** | **CC-09** | **CC-10** | **BC** |
| --- | --- | --- | --- | --- | --- | --- | --- | --- | --- |
| 7/12/17 | 770 |  | 210 | 816 |  |  |  |  |  |
| 8/14/17 | 204 |  | 80 | 435 |  |  |  |  |  |
| 9/19/17 | 82 |  | 55 |  | 88 | 276 |  |  |  |
| 11/1/17 | 31 |  | 37 |  | 66 | 57 |  |  |  |
| 5/16/18 | 126 |  | 63 |  | 86 | 31 |  |  |  |
| 6/13/18 | 1120 |  | 397 |  | 308 | 210 |  |  |  |
| 7/17/18 | 461 |  | 276 |  | 613 | 488 | 866 |  |  |
| 8/14/18 | 111 |  | 152 |  | 1414 | 56 | 272 |  |  |
| 8/27/18 | 1300 |  | 2419 |  | 1120 | 1986 | 2419 | 2419 |  |
| 10/15/18 | 112 |  | 59 |  | 59 | 127 |  |  |  |
| 11/28/18 | 21 |  | 38 |  | 8 | 16 |  |  |  |
| 6/3/19 | 66 |  | 68 |  | 68 |  | 50 |  | 34 |
| 7/22/19 | 344 |  | 197 |  | 410 |  | 205 |  | 249 |
| 8/20/19 | 161 | 20 | 121 |  | 248 |  | 160 |  | 93 |
| 9/23/19 | 613 | 649 | 517 |  | 411 |  | 179 |  | 517 |
| 5/13/20 | 21 |  | 57 |  | 15 |  | 11 |  | 24 |
| 6/27/20 | 119 |  | 144 |  | 56 |  | 69 |  | 74 |
| 7/22/20 | 238 |  | 186 |  | 961 |  | 387 |  | 206 |
| 8/20/20 | 59 |  | 58 |  | 75 |  | 33 |  | 167 |
| 8/26/20 |  | 2419 |  |  |  |  |  |  |  |
| 9/28/20 | 162 |  |  |  |  |  |  |  | 119 |
| 6/30/21 | 387 | 261 | 461 | 308 | 194 | 387 | 0 | 770 | 160 |
| 7/7/21 | 613 | 1300 | 241 | 2419 | 228 | >2419 | >2419 | 517 | 1733 |
| 7/13/21 | 195 | 2419 | 115 | 437 | 164 | 1011 | 437 | 534 | 326 |
| 7/21/21 | 131 | 131 | 112 | 613 | 102 | 111 | 54 | 219 | 138 |
| 7/27/21 | 167 | 345 | 139 | 345 | 133 | 261 | 96 | 345 | 365 |
| 8/4/21 | 148 | 345 | 517 | 326 | 285 | 236 | 26 | 921 | 147 |
| 8/11/21 | 866 | 411 |  | 649 | 435 | 1300 | 461 | 921 | 488 |
| 6/1/22 | 548 | 14 | 140 | 579 | 866 |  |  | 579 | 184 |
| 6/15/22 | 649 | 40 | 120 | 649 | 291 |  |  | 365 | 185 |
| 6/29/22 | 727 | 387 | 179 | 435 | 461 |  |  | 261 | 816 |
| 7/14/22 | 210 | 249 | 172 | 249 | 206 | 219 |  | 99 | 365 |
| 7/27/22 | 166 | 99 | 99 | 1553 | 308 | 488 |  | 192 | 387 |
| 8/10/22 | 199 | 72 | 148 | 210 | 135 | 206 |  | 219 | 291 |
| 8/24/22 | 102 | 77 | 91 | 166 | 192 | 161 |  | 121 | 107 |
| Note: Data presented are colony-forming units (CFU) with red cells indicating values over the single day water quality standard (WQS) of 300 CFU/100 mL *E. coli* results were obtained from SOS Analytical in Traverse City, MI. | | | | | | | | | |
